# Molecular and morphological analyses confirm *Rhizopogon verii* as a widely distributed ectomycorrhizal false truffle in Europe, and its presence in South America

**DOI:** 10.1007/s00572-015-0678-8

**Published:** 2016-01-14

**Authors:** Marcelo A. Sulzbacher, Tine Grebenc, Miguel Á. García, Bianca D. Silva, Andressa Silveira, Zaida I. Antoniolli, Paulo Marinho, Babette Münzenberger, M. Teresa Telleria, Iuri G. Baseia, María P. Martín

**Affiliations:** Departamento de Micologia/CCB, Universidade Federal de Pernambuco, Av. Prof. Nelson Chaves, s/n, CEP: 50670-901 Recife, Pernambuco Brazil; Slovenian Forestry Institute Večna pot 2, SI-1000 Ljubljana, Slovenia; Department of Biology, University of Toronto, 3359 Mississagua Road, Mississagua, ON L5L 1C6 Canada; Departamento de Botânica e Zoologia, Universidade Federal do Rio Grande do Norte, Campus Universitário, Lagoa Nova, CEP: 59072-970 Natal, Rio Grande do Norte Brazil; Departamento de Solos, Universidade Federal de Santa Maria, CCR, Campus Universitário, 971050-900 Santa Maria, Rio Grande do Sul Brazil; Departamento de Biologia Celular e Genética, Universidade Federal do Rio Grande do Norte, Campus Universitário, Lagoa Nova, CEP: 59072-970 Natal, Rio Grande do Norte Brazil; Institute for Landscape Biogeochemistry, Leibniz Centre for Agricultural Landscape Research (ZALF), Eberswalder Straße 84, 15374 Müncheberg, Germany; Departamento de Micología, Real Jardín Botánico, RJB-CSIC, Plaza Murillo 2, Madrid, 28014 Spain

**Keywords:** Boletales, Ectomycorrhiza, Hypogeous fungi, Internal transcribed spacer, nrDNA, *Pinus sylvestris*, *Pinus taeda*, Phylogeny

## Abstract

The genus *Rhizopogon* includes species with hypogeous or subepigeus habit, forming ectomycorrhizae with naturally occurring or planted pines (Pinaceae). Species of the genus *Rhizopogon* can be distinguished easily from the other hypogeous basidiomycetes by their lacunose gleba without columella and their smooth elliptical spores; however, the limit between species is not always easy to establish. *Rhizopogon luteolus*, the type species of the genus, has been considered one of the species that are more abundant in Europe, as well as it has been cited in pine plantation of North and South America, different parts of Africa, Australia, and New Zealand. However, in this study, based on molecular analyses of the ITS nuclear ribosomal DNA (nrDNA) sequences (19 new sequences; 37 sequences from GenBank/UNITE, including those from type specimens), we prove that many GenBank sequences under *R. luteolus* were misidentified and correspond to *Rhizopogon verii*, a species described from Tunisia. Also, we confirm that basidiomes and ectomycorrhizae recently collected in Germany under *Pinus sylvestris*, as well as specimens from South of Brazil under *Pinus taeda* belong to *R. verii*. Thanks to the numerous ectomycorrhizal tips collected in Germany, a complete description of *R. verii*/*P. sylvestris* ectomycorrhiza is provided. Moreover, since in this paper the presence of *R. verii* in South America is here reported for the first time, a short description of basidiomes collected in Brazil, compared with collections located in different European herbaria, is included.

## Introduction

The species of the genus *Rhizopogon* Fr. belong to the order Boletales and suborder Suillineae in the Agaricomycetidae (Binder and Hibbett 2006). The genus is represented with over 100 species distributed worldwide (Smith and Zeller 1966; Martín 1996; Martín and García 2009). All species produce hypogeous or semi-hypogeous basidiomes and form ectomycorrhizae (EcM) with members of the Pinaceae (*Pinus*, *Pseudotsuga*, and *Tsuga*). *Rhizopogon* species are easy to cultivate in pure culture (Molina and Trappe 1994; Brundrett et al. 1996); thus, some were frequently applied to study physiology, morphology, or ecology of its ectomycorrhizae in the agroforestry systems (Smith and Zeller 1966; Hung and Trappe 1983; Chu-Chou and Grace 1984; Miller 1986; Molina et al. 1997; Beiler et al. 2010).

Zeller and Dodge (1918) were the first authors to present a worldwide monograph of *Rhizopogon*. Later, Smith and Zeller (1966) produced the first modern account of the genus to North America including a total of 137 taxa, in which 128 were new for science. Since this paper, the Pacific Northwestern USA has been considered the greatest area of diversity of the genus (Hosford 1975; Molina et al. 1997; Grubisha et al. 2002), as well as other parts of the USA (Harrison and Smith 1968; Miller 1986). However, in posterior systematic studies undertaken in several part of the world, authors described new species in Mexico (Trappe and Guzmán 1971, Cázares et al. 1992), Tunisia (Pacioni 1984a), China (Liu 1985), Japan (Mujic et al. 2014), and different countries of Europe (Pacioni 1984b, Martín 1996, Martín and Calonge 2001); as well as new records, such as those of Mexico and Caribean countries (Hosford and Trappe 1980), Italy (Montecchi and Sarasini 2000), Japan (Hosford and Trappe 1988) and Spain (Martín and Calonge 2006), showing that the knowledge of the genus is not yet complete.

Nowadays, systematics and taxonomy of *Rhizopogon* have been under profound changes, mainly due to the use of molecular tools, specially using sequence-based analyses of the nuclear rDNA regions (nuc-ssu, nuc-lsu, ITS) and also mitochondrial genes (*atp*6, mt-lsu) (Grubisha 1998; Martín et al. 1998; Grubisha et al. 2002; Kretzer et al. 2003; Grubisha et al. 2005; Binder and Hibbett 2006; Martín and García 2009). According to Grubisha et al. (2002), the species are distributed in five subgenera: *Amylopogon*, *Rhizopogon*, *Roseoli*, *Versicolores*, and *Villosuli*. The species of the subgenus *Rhizopogon* have shown a combination of features, such as a simple peridium completely covered by rhizomorphs. *Rhizopogon luteolus* is the type species of the subgenus and it has been considered widely distributed in the Northern hemisphere.

*Rhizopogon verii* Pacioni (Pacioni 1984a) was described from Tunisia under *Pinus pinaster*. However, studies related to the systematic and distributions of *R. verii* are limited to only few collections from Italy, Spain, and Tunisia (Martín 1996). From other continents, *R. verii* has not been cited yet. Recent collections on an abandoned coal mine area near Crinitz (Brandenburg, Germany) on *Pinus sylvestris* could fit with *R. verii*, as well as the specimens collected during a survey of hypogeous fungi in State of Rio Grande do Sul (Brazil) growing under *Pinus taeda*.

Thus, with the opportunity to study new fresh specimens, the main objective of this paper was to clearly identify the specimens from Germany and Brazil using molecular analyses of ITS nrDNA sequences. This has allowed us also to confirm the presence of *R. verii* in these countries, as well as the EcM of *R. verii* on *P. sylvestris*. A detailed description is provided, both to the basidiomes and the EcM formed by *R. verii*/*P. sylvestris*. Moreover, information to *R. verii* worldwide distribution in different native and pine plantation areas is provided.

## Materials and methods

Specimens from Brazil were collected during mycological trips in the State of Rio Grande do Sul, close to the “Estação Ecológica do TAIM” in a sandy dune near to mature trees of *P. taeda*. In Germany, fresh basidiomes and soil cores to collect ectomycorrhizal tips were taken from the abandoned coal mine area along the side road toward Schlabendorfer See near the village Crinitz; the area is represented by a ca. 30-year-old *P. sylvestris* plantation established on silicate sandy neosol with shallow organic layer and poor understory vegetation. Data of new specimens and ectomycorrhiza collected for this paper are included in Table 1.

**Table 1:** Samples of *Rhizopogon verii* included in morphological and molecular analyses

Herbarium number or EcM code	Origin	Coordinates	Collection date	Host	Isolation source	Accession number
UFRN-fungos 2371	BR: Rio Grande do Sul, TAIM area	52° 31′ 43.4″ N	5 Jan 2012	*Pinus taeda*	Basidiomes	n.d.
		32° 32′ 05″ E				
UFRN-fungos 2372 (duplo URM 88223)	BR: Rio Grande do Sul, TAIM area	52° 31′ 4.4″ N	9 Jan 2013	*Pinus taeda*	Basidiomes	LN875275
		32° 32′ 05″ E				
LJF 4035	DE: Casel, Kozen	51° 67′ 88.27″ N	26 Sep 2014	*Pinus sylvestris* plantation	Basidiomes	LN875272
		14° 15′ 65.71″ E				
LJF 4003, LJF 4015, LJF 4022, LJF 4038, LJF 4039	DE: Crinitz, village of Bergen	51° 46′ 5.30″ N	19 Oct 2013	*Pinus sylvestris* plantation and natural regeneration	Basidiomes	LN875267 (LJF 4022)
		13° 44′ 46.22″ E				
LJF 4031	DE: Crinitz, NW from the village of Bergen	51° 76′ 68.35″ N	22 Sep 2014	*Pinus sylvestris* young plantation	Basidiomes	n.d.
		13° 74′ 46.97″ E				
LJF 4029	DE: Crinitz, NW from the village of Bergen	51° 76′ 71.30″ N	22 Sep 2014	*Pinus sylvestris* young plantation	Basidiomes	LN875271
		13° 74′ 49.02″ E				
LJF 4027	DE: Crinitz, NW from the village of Bergen	51° 76′ 66.19″ N	22 Sep 2014	*Pinus sylvestris* young plantation	Basidiomes	n.d.
		13° 74′ 45.91″ E				
LJF 4030, LJF 4058	DE: Gorden-Staupitz, Senftenberg strasse	51° 52′ 84.04″ N	24 Sep 2014	*Pinus sylvestris* plantation	Basidiomes	LN875273, LN875274
		13° 65′ 54.99″ E				
LJF4019, LJF 4036	DE: Göritz, Drebkau	51° 66′ 40.91″ N	26 Sep 2014	*Pinus sylvestris* plantation and *Alnus glutinosa*	Basidiomes	n.d.
		14° 10′ 77.43″ E				
LJF 4016, LJF 4055 (A), LJF 4055 (B), LJF 4055 (C)	DE: Hennersdorf	51° 38′ 7.43″ N	21 Oct 2013	*Pinus sylvestris* plantation with individual *Betula pendula*, *Robinia pseudoacacia*, *Quercus robur*, and *Q. rubra*	Basidiomes	LN875268, LN875264, LN875265, LN875266
		13° 37′ 31.10″ E				
LJF 4025, LJF 4032, LJF 4037, LJF 4041	DE: Hennersdorf	51° 63′ 54.69″ N	23 Sep 2014	*Pinus sylvestris* plantation with *Robinia pseudoacacia*	Basidiomes	LN875269 (LJF 4025), LN875270 (LJF 4032)
		13° 62′ 43.10″ E				
LJU-SFI-PSyl-2-2-1, LJU-SFI-PSyl-2-2-2, LJU-SFI-PSyl-2-2-3, LJU-SFI-PSyl-2-3-1, LJU-SFI-PSyl-2-3-3	DE: Hennersdorf	n.d.	n.d.	*Pinus sylvestris* plantation	Root tips	LN875259, LN875260, LN875261, LN875262, LN875263
LJF 4014	DE: Leippe	51° 25′ 24.36″ N	24 Oct 2014	*Pinus sylvestris* young plantation	Basidiomes	n.d.
		14° 2′ 52.61″ E				
LJF 4026, LJF 4042	DE: Lugkteich (lake), Lower Lusatian Ridge Nature Park	51° 72′ 38.07″ N	27 Sep 2014	*Pinus sylvestris* young plantation	Basidiomes	n.d.
		13° 58′ 85.12″ E				

*BR* Brazil, *DE* Germany, *n.d*. no data

### Morphological analyses

Fresh basidiomata were collected and analyzed macro- and microscopically following previously described methods (Miller and Miller 1988; Martín 1996), and compared with *R. verii* collections located at AQUI herbarium, including the type, as well as collections in BCN herbarium. Color codes followed Munsell Soil Color Charts (2009). Presentation of basidiospore data follows the methodology proposed by Tulloss et al. (1992), slightly modified by Wartchow (2012) and Wartchow et al. (2012). Abbreviations include *L*(*W*) = average basidiospore length (width), *Q* = the length to width ratio range as determined from all measured basidiospores, and *Q*_m_ = the *Q* value averaged from all basidiospores measured. Herbarium abbreviations follow those of the online version of Thiers [continuously updated]. Specimens are deposited in UFRN, URM and LJF herbaria.

Soil was gently washed from ectomycorrhizae (EcM) under binocular using forceps and brush, and subsequently EcM were stored in 2 % glutaraldehyde in 0.1 M sodium cacodylate buffer (pH 7.2) at room temperature. For semi-thin sections of mycorrhizae, six washes (10 min each) in 0.1 M sodium cacodylate buffer were performed. Samples were postfixed in 1 % osmium tetroxide in the same buffer for 1 h in the dark under room temperature. After six washes with distilled water, samples were dehydrated in acetone (25, 50, 70, and 95 %, for 15 min each) and three times in 100 % acetone for 1 h. The mycorrhizal tips were embedded in Spurr’s plastic (Spurr 1969) and sectioned with a diamond knife on an Ultracut Reichert Ultramicrotome (W. Reichert-LABTAC, Wolfratshausen, Germany). The sections (0.5 μm thin) were stained with crystal violet. Twenty mycorrhizal tips were investigated by the use of a light microscope (Axioscop 50, Zeiss, Oberkochen, Germany).

Macroscopic, anatomorphic, and biochemical characteristics were assessed as described in Agerer (1991), following also the computer character checklist from Agerer (1987–2012). A stereomicroscope (Zeiss SteREO Lumar.V12) with ×6.4–×80 magnification (Zeiss, Jena, Germany) and a microscope (Zeiss AXIO Imager.Z2) equipped for VIS, DIC, dark field, and fluorescent microscopy with magnification ×12.5–×1000 (Zeiss, Jena, Germany) were used to assess characters and make photos.

### DNA extraction, amplification, and sequencing

Total genomic DNA was extracted from the gleba of air-dried basidiomes or from stored ectomycorrhizal root tips (5–10 tips from the same cluster per extraction) by using a Plant DNeasy Mini Kit (Qiagen, Hilden, Germany). Extracted DNA was resuspended in pre-warmed, sterile Milli-Q water to the approximate final concentration of 100 ng μl^−1^ and kept at −80 °C. Primer pair ITS1F (Gardes & Bruns 1993) and ITS4 (White et al. 1990) was used for PCR amplification of the complete nuclear ITS region. Amplification reactions were performed in a PE 9700 DNA thermocycler, with an annealing temperature of 55 °C. Negative controls, lacking fungal DNA, were run for each experiment to check for any contamination. Amplified DNA was separated and analyzed as described in Grebenc et al. (2009).

Amplified DNA fragments were first separated and purified from the agarose gel using the Wizard SV Gel and PCR Clean-Up System (Promega Corporation, Madison, WI, USA) and sent to Macrogen Korea (Seoul, Korea) for sequencing. Sequencher 5.1 (Gene Codes Corporations, Ann Arbor, MI, USA) was used to identify the consensus sequence from the two strands of each isolate.

### Molecular analyses

Preliminary identification of the new sequences obtained were done through UNITE database (http://unite.ut.ee) species hypothesis (SH) search (Kõljalg et al. 2013). The PlutoF multiple sequence alignments obtained in UNITE were merged and manually adjusted using Se-Al v.2.0a11 (Rambaut 2002). The sequence AF062933 of *Rhizopogon succosus* A.H. Sm. was chosen as outgroup, since it is one of the few sequences available of subgen. *Roseoli* Fr. with voucher collection, excluding the sequences of *R. luteolus* and *R. verii*.

Analyses were conducted using parsimony and Bayesian inference. In the parsimony analyses, nucleotide characters were treated as unordered and all changes were equally weighted; gaps were treated as missing data. Searches for most parsimonious (MP) trees were performed using a two-stage strategy with PAUP* v.4.0b10 (Swofford 2002). First, the analyses involved 10,000 replicates with stepwise random taxon addition, tree bisection-reconnection (TBR) branch swapping saving no more than 10 trees per replicate, and MULTREES option off. The second round of analyses was performed on all trees in memory with the same settings except the MULTREES option on. Both stages were conducted to completion or until one million trees were found. Relative support for clades was inferred by nonparametric bootstrapping (Felsenstein 1985) as implemented in PAUP* using 500 pseudoreplicates, each with 20 random sequence addition cycles, TBR branch swapping, and MULTREES option off (DeBry and Olmstead 2000). To the Bayesian analyses, the program MrModeltest v.2.3 (Nylander 2004) was used to determine the model of sequence evolution that fits best the dataset. The Hasegawa-Kishino-Yano (Hasegawa et al. 1985) of DNA substitution, with rate variation among nucleotides following a discrete gamma distribution (HKY + G), was selected as the best-fit by both the hierarchical likelihood ratio test (hLRT) and Akaike information criterion (AIC). Bayesian phylogenetic inferences were performed using MrBayes v.3.2.2 (Ronquist et al. 2012) run on the CIPRES Science Gateway (Miller et al. 2010). Two runs starting from random trees were carried out using the HKY + G substitution model. All model parameters were treated as unknown variables with uniform prior probabilities and were estimated as part of the analysis together with tree topologies. Metropolis-coupled Markov chain Monte Carlo algorithm was used with eight simultaneous chains for each run, set at two million generations, and sampled every 1000 generations. Of the 40,002 trees obtained, the first 25 % were discarded as burn-in; the 50 % majority-rule consensus tree and the Bayesian posterior probabilities (PP) were obtained in MrBayes from the remaining 30,002 trees.

## Results

### Molecular analyses

**Fig. 1:**
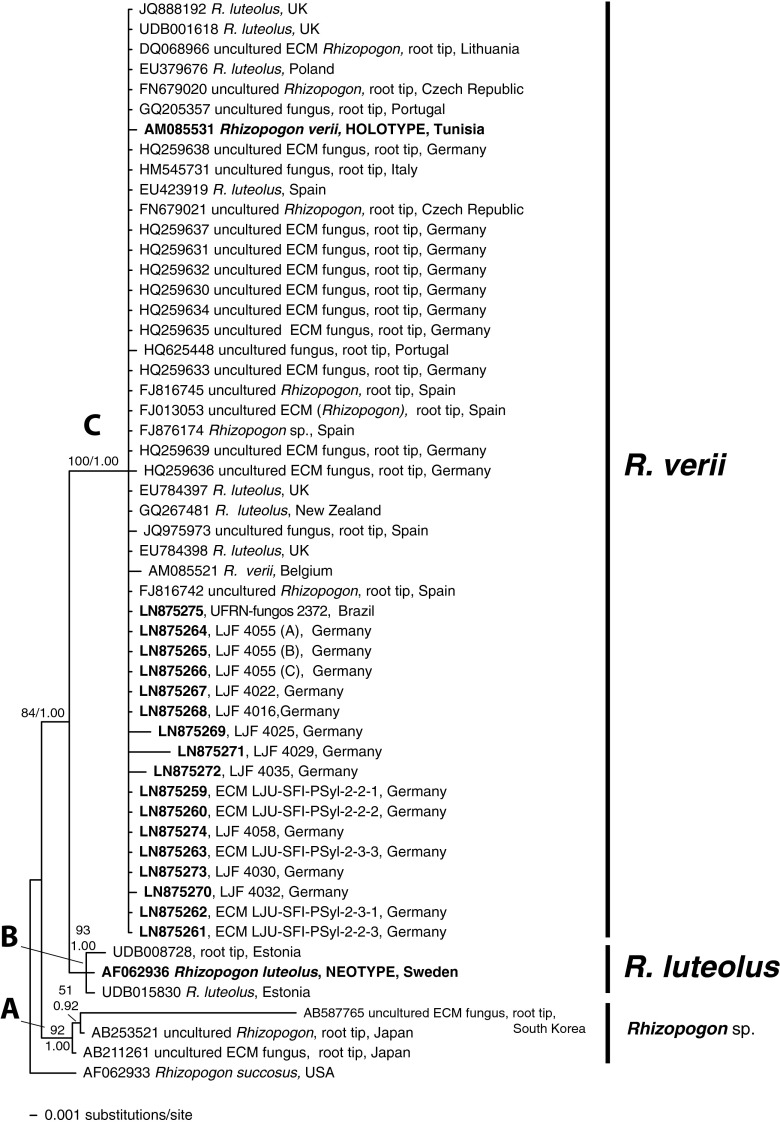
The 50 % majority-rule consensus tree of ITS nrDNA sequences of *Rhizopogon luteolus* and *R. verii* using Bayesian approach. A sequence of *R. succosus* was indicated as outgroup. Sequences from *Rhizopogon luteolus* and *R. verii* specimen types are marked in *bold*, as well as the accession numbers of the new sequences obtained in this study from Brazil and Germany. *Numbers at the nodes* indicate the percentage of boostrap values obtained from parsimony analysis with PAUP, and the posterior probabilities from the Bayesian analysis

The matrix contained the 19 sequences obtained in this study (Table 1) and sequences of the species hypothesis groups SH5_008910 and SH5_008911 obtained through UNITE search (Table 2: SH5_008910, clade A and C; SH5_008911, clade B). After manual adjustment, the matrix had 749 characters, 95 of them variable and 23 parsimony-informative that produced >1,000,000 MP trees, 107 steps in length. There was a consistency index of 0.953 and a retention index of 0.941. The harmonic mean of the estimated marginal likelihoods from the Bayesian analysis was −ln = 1727.88. The MP and Bayesian analyses produced trees of identical topology (Fig. 1), representing the Bayesian Majority Rule Consensus tree with the PP and Bootstrap values on the branches.

**Table 2:** Metadata from NCBI and UNITE sequences included in the molecular analyses

Clades/Taxon names	Acc. Number NCBI or UNITE	Sequence name in databases	Isolation source	Origin	Host	Publication were the sequences Unpublished
Clade A *Rhizopogon* sp.	AB211261	Uncultured ECM fungus	Root tip	Japan	*Pinus densiflora*	Lian et al (2006)
	AB253521	Uncultured *Rhizopogon*	Root tip	Japan:Tottori, Tottori sand dune	*Pinus thunbergii*	Taniguchi et al (2007)
	AB587765	Uncultured ECM fungus	Root tip	South Korea: Kangwon-do	*Pinus thunbergii*	Obase et al (2011)
Clade B *Rhizopogon luteolus* Fr. & Nordhom	AF062936, neotype	*R. luteolus*	Basidiome	Sweden: Uppsala	*Pinus* sp.	Grubisha et al (2002)
	UDB008728	ECM *Suillus*-*Rhizopogon* clade	Root tip	Estonia: Kuusnõmme	*Pinus sylvestris*	Unpublished
	UDB015830	*R. luteolus*	Basidiome	Estonia: Audaku, Saare	Mixed forest	Unpublished
Clade C *Rhizopogon verii* G. Pacioni	AM085521	*R. verii* (under *R. corsicus* in herbarium label)	Basidiome	Belgium: Limburg	Probably planted pines from Corsica	Unpublished
	AM085531, Holotype	*R. verii*	Basidiome	Tunisia: Tabarka	*Pinus pinaster*	Martín and García (2009)
	DQ068966	Uncultured ECM *Rhizopogon*	Root tip	Lithuania	*Pinus sylvestris*	Menkis et al (2005)
	EU379676	*R. luteolus*	Root tip	Poland	*Pinus sylvestris*	Hilszczanska et al (2008)
	EU423919	*R. luteolus*	Basidiome	Spain	*Pinus pinea*	Hortal et al (2008)
	EU784397	*R. luteolus*	Basidiome	UK: Surrey	–^a^	Brock et al (2009)
	EU784398	*R. luteolus*	Basidiome	UK: South Hampshire	–^a^	Brock et al (2009)
	FJ013053	Uncultured ECM (*Rhizopogon*)	Root tip	Spain	*Pinus pinaster*	Rincón & Pueyo (2010)
	FJ816742, FJ816745	Uncultured *Rhizopogon*	Root tip	Spain	*Pinus pinaster*	Pestana & Santolamazza (2011)
	FJ876174	*Rhizopogon* sp.	Root tip	UK: England, Stoborough Heath National Nature Reserve	*Pinus* sp.	Collier & Bidartondo (2009)
	FN679020	Uncultured *Rhizopogon*	Root tip	Czech Republic: Bohemian Switzerland National Park	*Pinus sylvestris*	Kohout et al (2011)
	FN679021	Uncultured *Rhizopogon*	Root tip	Czech Republic: Bohemian Switzerland National Park	*Pinus strobus*	Kohout et al (2011)
	GQ205357	Uncultured fungus	Root tip	Portugal	*Pinus pinaster*	Buscardo et al (2010)
	GQ267481	*R. luteolus*	Basidiome	New Zealand	*Pinus radiata*	Walbert et al (2010)
	HM545731	Uncultured fungus	Root tip	Italy	*Pinus pinaster*	Buscardo et al (2011)
	HQ259630-HQ259639	Uncultured ECM fungus	Root tip	Germany: Saxony-Anhalt, Duebener Heide, Roesa	*Pinus sylvestris*	Schulz et al (2012)
	HQ625448	Uncultured fungus	Root tip	Portugal	*Pinus pinaster*	Buscardo et al (2012)
	JQ888192	*R. luteolus*	Basidiome	UK: Scotland, Culbin forest	*Pinus sylvestris* and *P. nigra* plantation	Pickles et al (2012)
	UDB001618					
	JQ975973	Uncultured fungus	Root tip	Spain	*Pinus pinaster*	Rincón et al. (2014)
Outgroup	AF062933	*Rhizopogon succosus*	Basidiome	USA: West Virginia	*Pinus* sp.	Grubisha et al (2002)

^a^Unknown possible host

Including *R. succosus* as outgroup, sequences are distributed in three highly supported clades. The clade A (bs = 92 %, pp = 1.0) grouped three sequences from Japan and South Korea, collected under *Pinus densiflora* and *Pinus thunbergii* from unidentified collections (both basidiomata and ECM). The clade B (bs = 93 %, pp = 1.0) included three sequences, two from Estonia and the sequence from the neotype of *R. luteolus* from Uppsala (Sweden) [designated in Martín (1996)], all under *Pinus* species; this *R. luteolus* clade is the sister group of the clade C (bs = 84 %, pp = 1.0) that grouped 47 sequences, including the sequence of the type of *R. verii*, a species described from Tunisia under *P. pinaster*, eight sequences identified as *R. luteolus* collected under different *Pinus* species (mainly *P. pinaster* and *P. sylvestris*), from Europe and New Zealand, and many sequences from uncultured ectomycorrhizal fungi. All new sequences obtained from Germany and Brazil were grouped in clade C, confirming that they belong to the species *R. verii*.

### *Rhizopogon verii* morphological descriptions

**Fig. 2:**
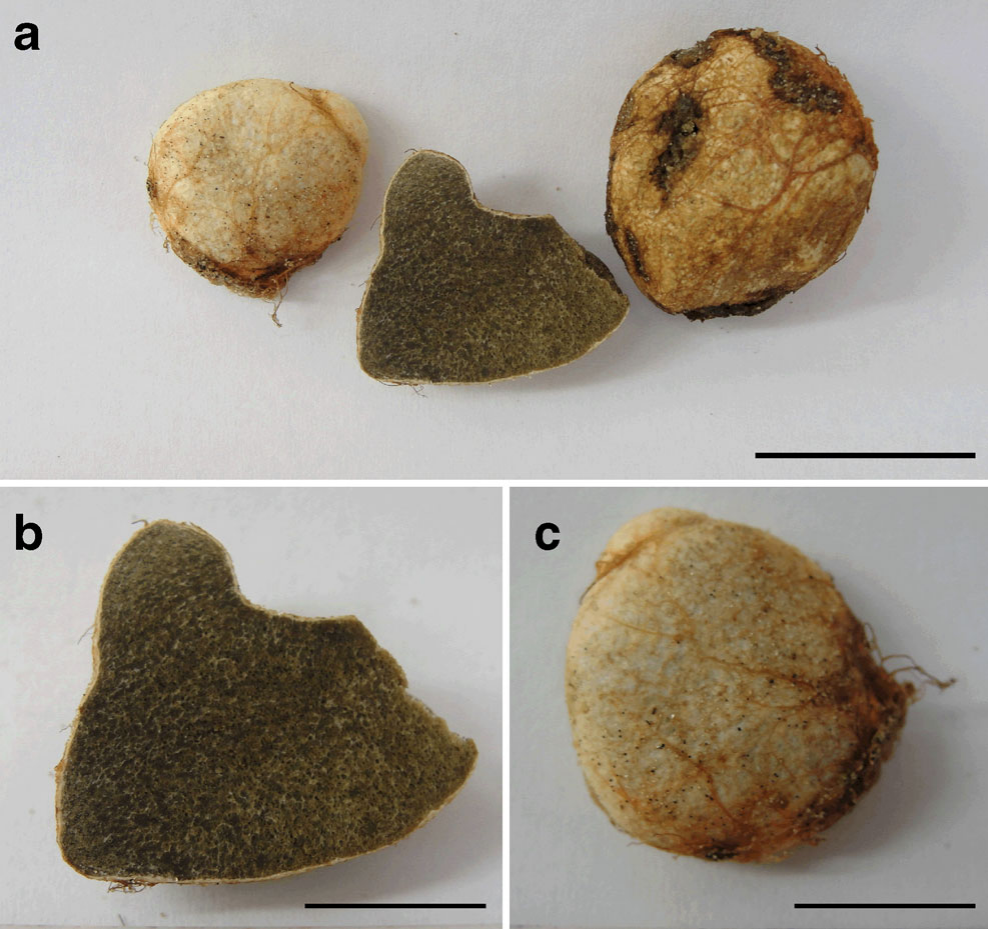
*Rhizopogon verii* (UFRN-fungos 2372). **a** Basidiomes. **b** Longitudinal section of basidioma showing the gleba. **c** Surface of peridium showing the reddish rhizomorphs. *Scale bars* represent 20 mm (**a**) and 10 mm (**b**–**c**)

**Fig. 3:**
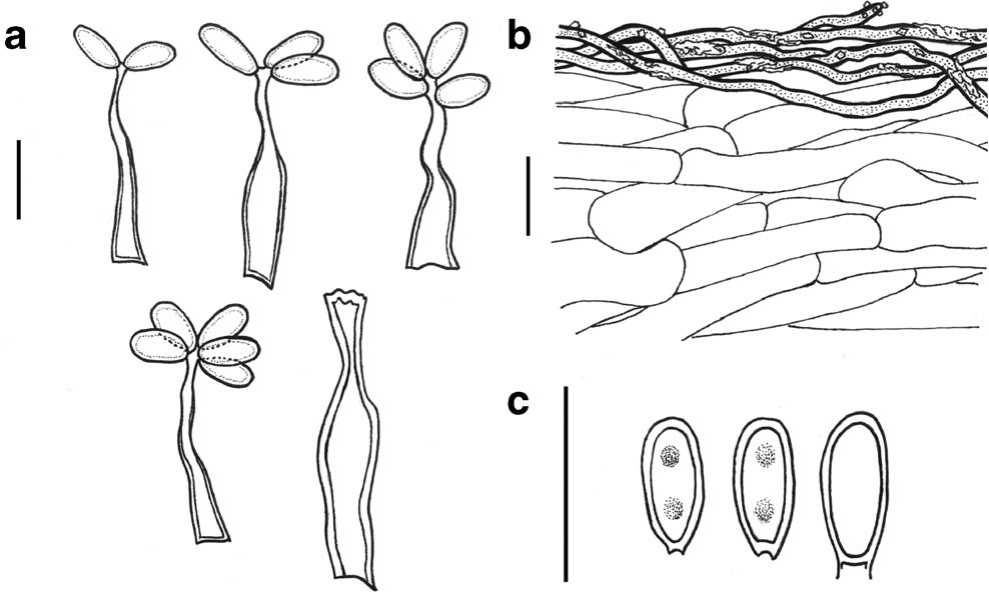
*Rhizopogon verii* (UFRN-fungos 2372). **a** Basidia. **b** Peridium showing differentiation of the outer and inner peridial layers. **c** Basidiospores. *Scale bars* represent 10 μm

*Basidiomes* (7–) 18–23 mm width, (11–) 20–27 mm high, depressed subglobose to irregular, others are compressed, covered by red to reddish yellow rhizomorphs (HUE10R 5/8), 0.1–05-mm diam., appressed to the peridium (Fig. 2a, c). Peridium <0.5 mm thick, pink (HUE7.5YR 8/4) to reddish yellow (HUE 7.5YR 7/6) in maturity, glabrous. Gleba loculate, rounded locules up to 0.5-μm diam., none gelatinized, olive brown (HUE 2.5Y 4/4) to dark-reddish-brown (HUE 2.5YR 3/4) at maturity, columella absent (Fig. 2a, b). *Microscopic characters*: Peridium 358–384 μm thick, composed of prostate to interwoven hyphae (*luteolus*-type); external layer formed by abundant yellowish brown to brown hyphae, walls thin to thickened, encrusted with irregular granules and crystals, some amorphous, brown pigmented bodies also present, 1.5–7-μm diam; internal layer composed by hyaline, smooth, and thick-walled hyphae, compactly interwoven, filamentous to inflated hyphae broader than the external layer, 3–12-μm diam. (Fig. 3b). Trama 11–25 μm thick, formed by interwoven hyphae, often in part gelatinized, hyaline, smooth and thin-walled, simple septate hyphae, 1–5-μm diam. Clamp connections absent in all septa. Subhymenium ramose, hyaline, 3–5-μm diam. Brachybasidioles clavate to cylindrical (12–) 14–20 × 3–5 μm. Basidia are lageniform, with a thick-walled (<1.5-μm diam.), ventricose base (9–20-μm length × 3.5–8 μm width), and a thin-walled beak (5.5–14.5 μm length × 2–4 μm width), developing from 6 to 8 hyaline sterigmata (Fig. 3a). Basidiospores 5–8 × 2–3 μm (*L* = 6.6 μm, *W* = 2.3 μm, *Q* = 2–3.5 (– 4.5), *Q*_m_ = 2.94), narrowly ellipsoid, elongate to slightly cylindrical, with a not much truncate apex, smooth and thin to thickened wall, hyaline to pale greenish in KOH 5 %, generally mono- or bi-guttulate (Fig. 3c). *Chemical reactions*: Peridium with KOH 5 % revives orange pigments, even in dried specimens.

**Fig. 4:**
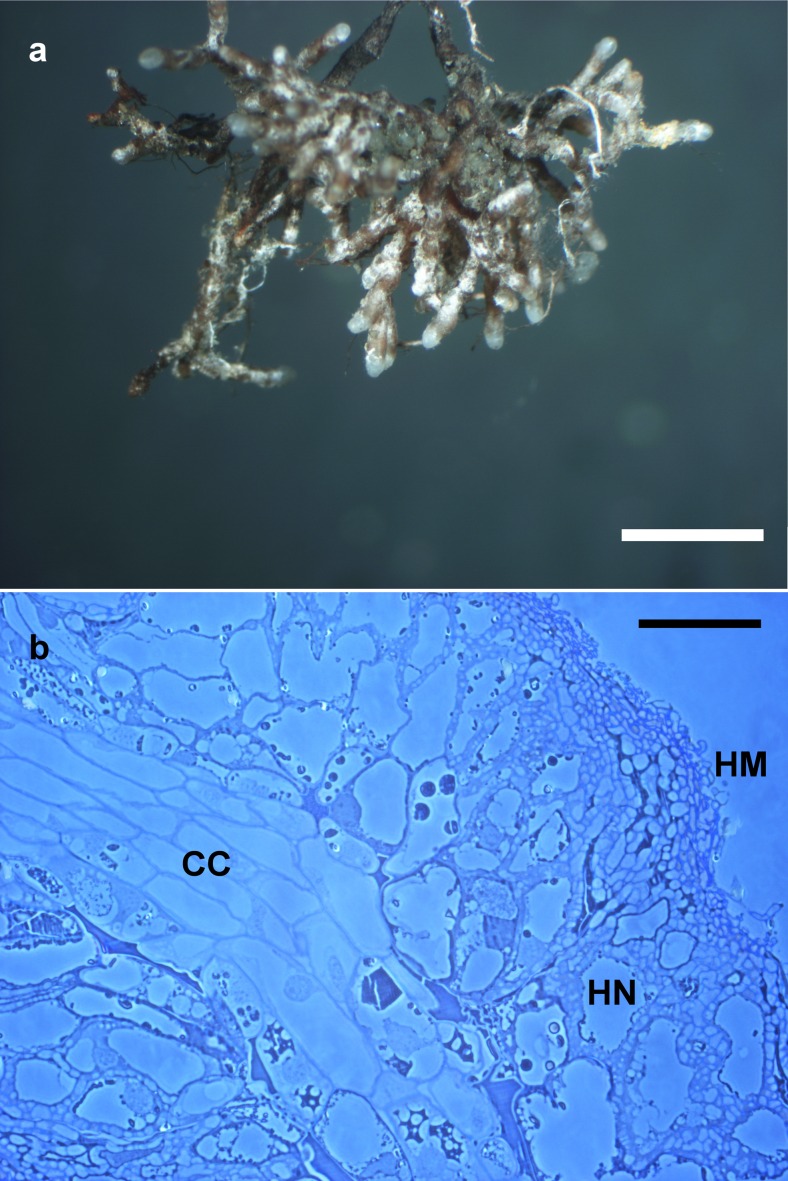
Ectomycorrhiza *Rhizopogon verii*-*Pinus sylvestris*. **a** Habitus. **b** Longitudinal semi-thin section of the ectomycorrhiza *Rhizopogon verii*-*Pinus sylvestris. CC* central cylinder, *HM* hyphal mantle, *HN* Hartig net. *Scale bars* represent 2 mm (**a**) and 50 μm (**b**)

**Fig. 5:**
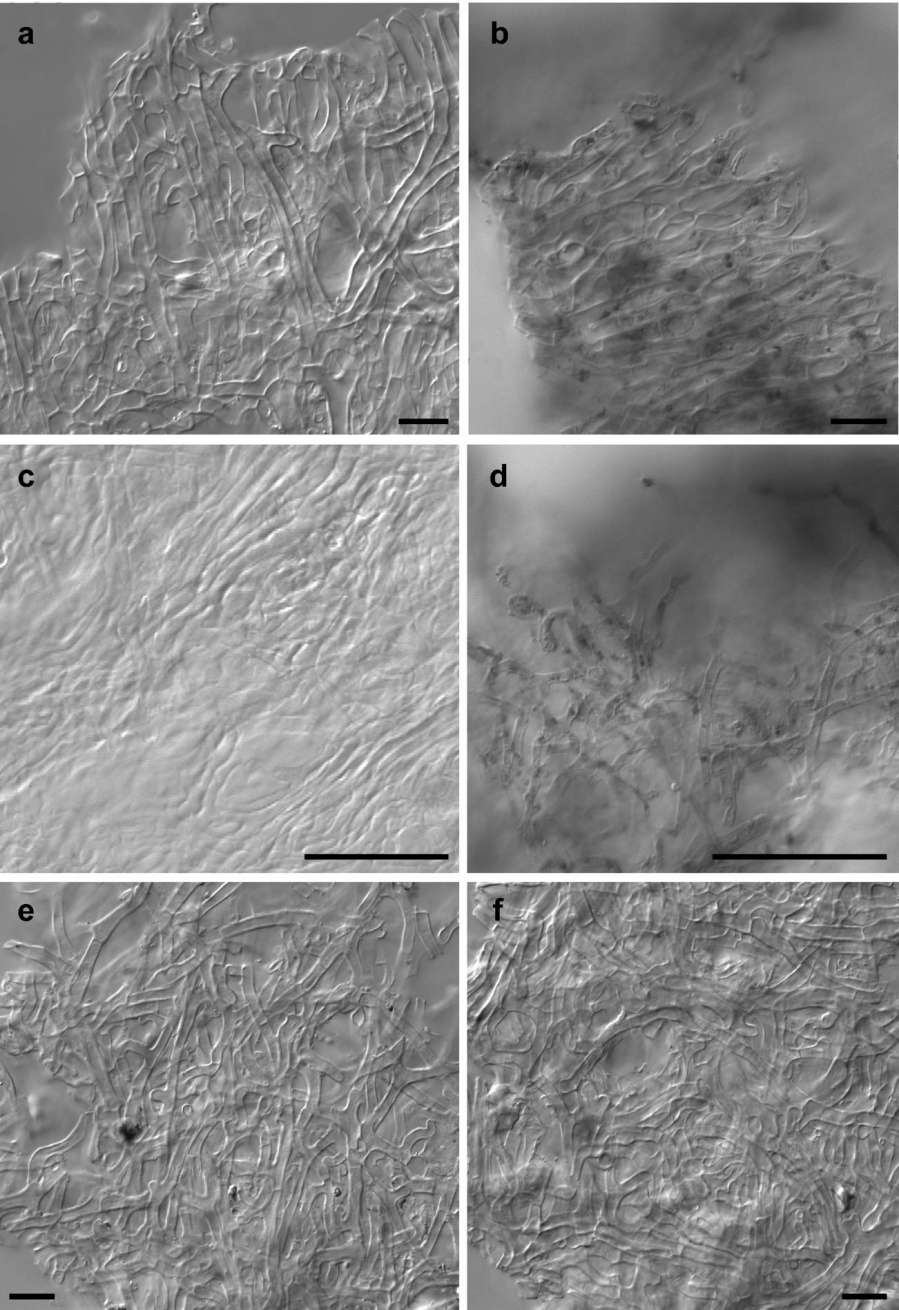
Anatomy of *Rhizopogon verii* ectomycorrhiza. **a** The outer mantle layers with septated, occasionally branched, and at distal ends inflated hyphae. **b** The middle mantle layers with hyphae arranged in broad streaks of parallel hyphae and gelatinous matrix present. **c** The pseudoparenchymatous inner mantle layers, hyphae arranged with no pattern and gelatinous matrix present. **d** Hyphal net over ectomycorrhiza. **e** Outer mantle layers. **f** Inner mantle layers of the very tip of ectomycorrhiza. *Scale bars* represent 10 μm (**a**, **b**, **e**, **f**) and 50 μm (**c**, **d**)

**Fig. 6:**
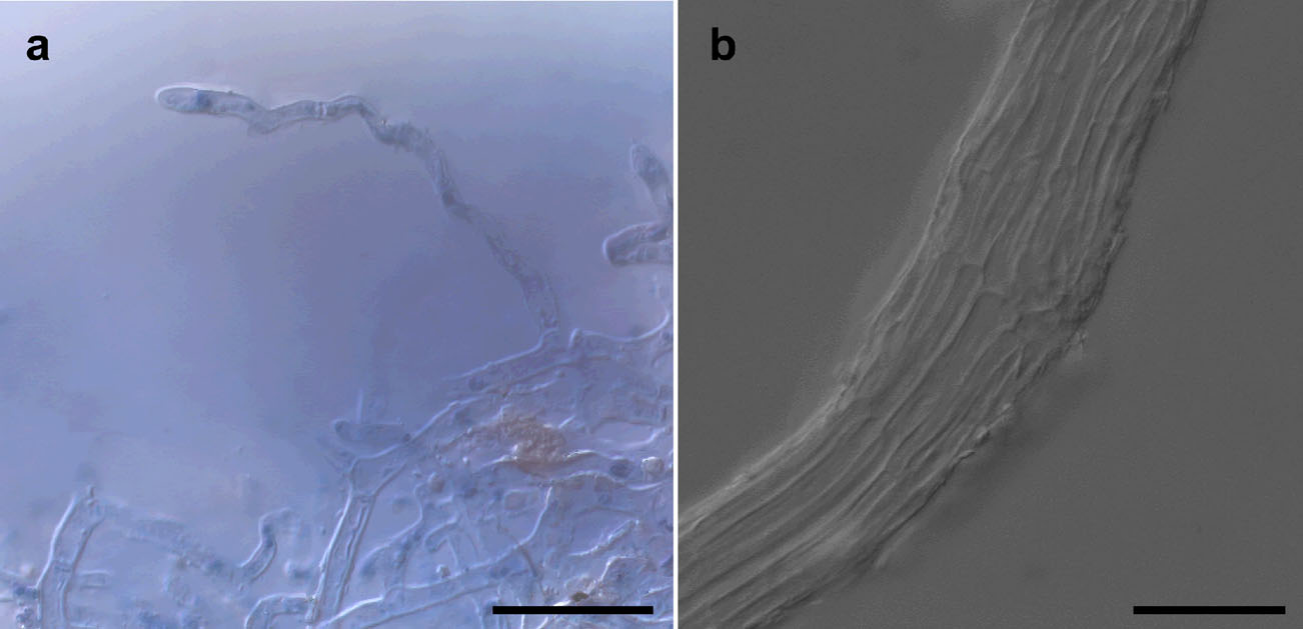
Anatomy of *Rhizopogon verii* ectomycorrhiza emanating elements. **a** Cystidia in the form of a normal hypha but twisted (type L). **b** Rhizomorphs differentiated with thick central hyphae and complete septa (type E). *Scale bars* represent 20 μm (**a**) and 10 μm (**b**)

*Ectomycorrhiza* (Fig. 4a) dichotomous ramified with 1–4 orders; ectomycorrhizal systems dense and abundant; distinct mantle surface and cortical cells not visible; mantle not transparent, mycorrhiza surface reticulate, taste mild, surface hydrophobic; system 1–10 mm long, unramified ends <2 mm long, diameter of unramified ends 0.20–0.40 μm; mycorrhizal ends straight, not inflated. Ectomycorrhiza ochre to yellowish, in parts shiny, older parts ochre to yellowish covered with soil particles; the very tips ochre to yellowish, no soil particles attached; older parts light brown, shiny, not carbonizing, no dots on mantle. *Laticifers* absent. *Rhizomorphs*—present, infrequent, origin proximal with a distinct connection to mantle; infrequently ramified, at restricted point; concolors to mantle (ochre, yellowish brown); margin smooth, in cross-section roundish, 5–60 μm in diameter, emanating hyphae present but infrequent; sclerotia on rhizomorphs not observed. *Anatomy of outer mantle layers* (Fig. 5a): plectenchymatous, hyphae rather irregularly arranged, no special pattern discernible (type B); hyphae with septae, forked, some hyphal junctions inflated at distal end; cells 10–50 (80)-μm long, 2–7 μm in diameter; hyphal net present, loose, some terminal hyphae forming cystidia; cells not filled with oily droplets, drops of exuded pigment, brownish content or needle-like content, blue granules, crystals, or cells of mounds absent; cell not colored, cell walls thin (<2 μm), cells 3–7 μm in diameter; clamps absent, septa as thick as walls, surface of cells smooth; matrix not gelatinous. *Anatomy of middle mantle layers* (Fig. 5b) plectenchymatous, hyphae arranged in broad streaks of parallel hyphae, matrix present and gelatinous; cells (5) 8–30 (80) μm long, 2–5 (7) μm in diameter; cells not filled with oily droplets brownish content or needle-like content, blue granules, crystals, or cells of mounds absent; cell not colored. *Anatomy of inner mantle layers* (Fig. 5c): pseudoparenchymatous, hyphae arranged with no pattern, matrix present and gelatinous; clamps not observed; cells not filled with oily droplets, brownish content or needle-like content and blue granules not observed. *Anatomy of outer mantle layer of ectomycorrhizal tip* (Fig. 5e, f): organized like other parts of mantle. *Anatomy of cystidia* (Fig. 6a): cystidia present, infrequent, only one type of cystidia present in the form of a normal hypha but twisted (type L); cells with septa, septa simple, no clamp connection observed, thin walled, cell walls not colored; cells not filled, no apical knob present, not branched; cells 10–50 (–65) μm long, diameter of proximal ends 3–6 μm; and distal ends 2–5 μm; surface smooth or infrequently covered with soil particles. *Anatomy of emanating hyphae* (Fig. 5d): hyphae observed as hyphal net over ectomycorrhiza forming short non-branched or branched terminal hyphae but not forming cystidia; cell walls thin, not colored, or infrequently covered with soil particles; clamp connections not present; anastomoses present, infrequent, opened with a long or rarely short bridge, anastomose bridge as thick as hyphae, cell walls of anastomoses as thick as hyphae. *Anatomy of rhizomorphs* (Fig. 6b): differentiated with thick central hyphae and complete septa (type E); nodia present, conical young side branches lacking, gelatinous matrix lacking, trumpet-like ambulate hyphae present; cystidia, laticifers, surface cell staining with sulfo-vanilline and hyphae filled with brownish substance or crystal-like reflecting content, blue granules all absent; central vessel-like hyphae present, without or with one side branch at septum, diameter 6–8 (–10) μm, thickened part distal, cell wall thin and color of cell walls lacking; non-vessel-like central hyphae 2–5 (–6) μm in diameter, central hyphae with septa, no clamp connections observed, septa of the same thickness as walls, color of cells lacking; peripheral hyphae 2–5 (–7) μm in diameter, cell walls <1 μm thick, surface smooth, droplets of secreted pigment, color of cells, balls of intertwined ramified thin hyphae or crystals all lacking. *Chlamydospores* not observed. *Sclerotia* not observed. *Anatomy of longitudinal section* (Fig. 4b): mantle 50–100 μm thick, different layers in mantle discernable, outer mantle layer plectenchymatous, inner mantle layer pseudoparenchymatous. *Hartig net* palmetto type with a single hyphal row (Fig. 4b), no haustoria observed. *Autofluorescence*: of the whole mycorrhiza not observed for rhodamine, green fluorescent, and DAPI filters. *Chemical reactions*: sulfo-vanilline—no reaction; lactic acid—no reaction, cotton blue lactic acid—blue spots in mantle cells.

Distribution: Belgium, Brazil, Czech Republic, Germany, Lithuania, New Zealand, Spain, Tunisia, and the UK.

Specimens examined: The data of the specimens examined are included in Table 1. Brazilian collections are located at the herbarium of the Universidade Federal do Rio Grande do Norte of (UFRN) and Universidade Federal de Pernambuco (URM), and the German specimens at the Mycotheca and Herbarium of Slovenian Forestry University (LJF).

## Discussion

*Rhizopogon verii* was originally described from Tunisia (Pacioni 1984a), under *P. pinaster*. Since Pacioni’s discovery, little was published related to these species; however, in the past few years, new samples were collected in Spain (Martín 1996). With the data obtained in our study, a general distribution of *R. verii* is shown, both in natural and planted pine forests, and it is expected that this species can be found in other countries where pine plantations were established using European seedling material.

Based on morphological data, Martín (1996) considered that in Europe, the wider distributed *Rhizopogon* species with *luteolus*-type peridium was *R. luteolus*. However, the present study combining basidiomata and EcM anatomy, together with molecular analyses, shows that the species *R. verii* is well defined and commonly present in Europe. Distribution of *R. verii* on other continents is fairly unknown, but the ecology of known sites indicates several similarities. Mineral and sandy soil requirements (Table 1) with Pinaceae for *Rhizopogon* were recorded for Europe and Africa (Raidl and Agerer 1998). Similarly the Brazilian specimens were gathered on sandy soil in natural sand dune plots, at the base of a *P. taeda* site in the Campos Sulinos (or Pampa) biome, especially covered by open grassy formations used as natural pastures (Overbeck et al. 2007; Fiaschi and Pirani 2009). In a floristic study for Brazil, Porto and Dillenburg (1986) reported that the indigenous vegetation is composed by members of Bignoniaceae, Cactaceae, Euphorbiaceae, Fabaceae (subfam. Caesalpinoideae and Faboideae), Myrtaceae, Nyctaginaceae, Rubiaceae, and Sapotaceae, but the presence of exotic tree species, such as *Pinus* or *Eucalyptus*, was required. *Rhizopogon* data from the tropical and subtropical region are rarely available, limiting the knowledge about the identification and phylogenetic placement of those fungi. In Brazil, the genus was introduced through seedlings of exotic *Pinus* spp. (Sulzbacher et al. 2013) in the southern and southeast region (Giachini et al. 2000; Baseia and Milanez 2002; Giachini et al. 2004; Sobestiansky 2005; Cortez et al. 2011). Neves and Capelari (2007) reported seven species of this genus in a Brazilian checklist (*R. fuscorubens* Smith, *R. luteolus* Fr. & Nordholm, *R. nigrescens* Coker & Couch, *R. roseolus* Corda sensu Smith, *R. rubescens* (Tul.) Tulasne, *R. vulgaris* (Vitt.) Lange, and *R. zelleri* Smith).

The studied *R. verii* Brazilian basidiomata covered different developmental stages (Fig. 2a–c), thus providing more information related to the basidiome morphology. The Brazilian collection exhibits basidiomata very similar to that illustrated by Pacioni (1988): subglobose to irregular basidiomata, covered by reddish yellow rhizomorphs and with an olive brown gleba. *Rhizopogon verii* shared several features with the widespread *R. luteolus* (Martín 1996), for example, the basidiome shapes, rhizomorphs covering the whole peridium surface, also the shape and size of basidiospores and the *luteolus*-type peridium. However, *R. luteolus* has a clavate to cylindrical basidia, with thin wall, and *R. verii* has mostly lageniform basidia, with a thick-walled ventricose base up to 1.5 μm diam. as described in Martín (1996). This morphological distinction between *R. luteolus* and *R. verii* is well supported by phylogenetic species delimitation using nrDNA ITS spacer molecular characterization which separated these two morphological groups in two distinct terminal clades (Fig. 1).

The comprehensive description of *R. verii* ectomycorrhiza on *P. sylvestris* is provided for the first time. In comparison to other described ectomycorrhizae from the genus *Rhizopogon* (www.deemy.de; Mohan et al. 1993), ectomycorrhiza of *R. verii* can be easily distinguished at least by the plectenchymatous mantle type B bearing some inflated cells at proximal end next to septae, the presence of rhizomorphs type E, cystidia type L, and loose hyphal net covering ectomycorrhiza. On the other hand, *R. luteolus* ectomycorrhiza showed type E of outer mantle layers, no emanating hyphae of cystidia and type F rhizomorphs (Uhl 1988). A more distant related *R. roseolus* ectomycorrhiza showed outer mantle type C and distinct reddish to whitish color of vital ECM tips (Raidl and Agerer 1998) and *Rhizopogon melanogastroides* with the same mantle type but ectomycorrhiza color similar yellowish to *R. verii* (Raidl et al. 1998). Morphological ECM characters of *R. verii* fit well to some previous observations by Agerer (2006) who noted that *Rhizopogon* has one of the most advanced rhizomorph-type structure (the boletoid rhizomorphs).

The combination of molecular analysis and morphological analyses of sporocarps and ectomycorrhiza supports the separation of *R. verii* from other *Rhizopogon* species. We also confirmed that *R. verii* has global distribution, most likely to originate from Europe but being introduced to all continents, with most recent discovery in South America, namely from pine plantation in Brazil and any further exploitations of the species globally would contribute valuable information to its distribution and ecology. As mentioned, in Tedersoo et al. (2010) and Tedersoo and Smith (2013), some lineages, such as the genus *Rhizopogon*, are restricted to this host family, hence explaining their geographical distribution.
